# Comparison of Conventional Bipolar Electrocautery and Ultrasonic Harmonic Scalpel in Colorectal Cancer Surgeries

**DOI:** 10.7759/cureus.23255

**Published:** 2022-03-17

**Authors:** Premchand Kalari, Dakshayani S Nirhale, Ramya Vajja, Pushkar Galam

**Affiliations:** 1 General Surgery, Dr. D.Y. Patil Medical College and Research Centre, Pune, IND; 2 Surgery, Dr. D.Y. Patil Medical College and Research Centre, Pune, IND

**Keywords:** operative time, blood loss, harmonic scalpel, electrocautery, colorectal cancer

## Abstract

Background and objective

Colorectal cancer (CRC) is the third most common malignancy and the second most deadly cancer worldwide. Powered equipment has transformed modern surgery, revolutionizing the delicacy, precision, and accuracy of many surgeries. The safety and efficacy of tissue dissection and artery sealing in colorectal surgery remain highly debatable. With the increased use of minimally invasive procedures in colon and rectal surgery, energy devices for tissue dissection and vascular sealing have become widely used. In light of this, we aimed at comparing the use of bipolar electrocautery and harmonic scalpel in CRC surgeries.

Methods

Our study was a hospital-based comparative study conducted at our tertiary care hospital. Fifty patients were divided equally into two groups by block randomization, and bipolar electrocautery was used in one group, and harmonic scalpel was used in the second group during surgery. The mean operative time, blood loss, and hospital stay were calculated in both groups. The comparison between bipolar electrocautery and harmonic scalpel was evaluated using independent t-tests.

Results

The mean operative time, blood loss, and hospital stay were significantly lower in the harmonic scalpel group than in the electrocautery group. The results were statistically significant (p < 0.001).

Conclusion

Based on our findings, the harmonic scalpel is a better energy source when compared to bipolar electrocautery in CRC surgeries.

## Introduction

Colorectal cancer (CRC) is the third most common malignancy and the second most deadly cancer worldwide [1]. According to Global Cancer Incidence, Mortality and Prevalence (GLOBOCAN) 2020 statistics, estimated new cases of CRC were 1.9 million, and estimated deaths were about 9,35,000, accounting for one-tenth of almost all cancer cases and fatalities in the year 2020 [2]. CRC is more common in industrialized nations, and its prevalence is rising in the middle- and low-income countries due to westernization. In addition, population ageing, poor dietary habits, smoking, lack of physical activity, and obesity contribute to the increased incidence of CRC [3].

Powered equipment has transformed modern surgery, revolutionizing the delicacy, precision, and accuracy of many surgeries. It may be tough to comprehend, but as energy becomes a cutting-edge technology, minimally invasive surgery becomes a reality and a valuable instrument in our doctors' tool belt. In 1920, William T. Bovie, an eccentric inventor, developed innovative electrosurgical equipment brought into clinical practice by Harvey Cushing, the creator of modern neurosurgery [4]. After 100 years, this essential instrument continues to be a critical tool in surgical practice. It is vital to prevent bleeding during general surgery when creating incisions and doing surgical dissection. Electrocautery is a well-established technique for haemostasis (the stopping of bleeding). Both the process and the tool used in operation are referred to as "electrocautery". However, electrocautery has several dangers, including more tissue damage than anticipated, haemostasis failure, and smoke generation. Newer alternative energy surgical devices are available and employed in a limited number of general procedures. Given the hazards involved with electrocautery, the use of alternative-energy devices may be a feasible option, but informed decision-making requires an awareness of the therapeutic efficacy of the newer technologies. A review of empirically supported recommendations will aid in the decision-making process. A harmonic scalpel is a surgical device that can cut and cauterize tissue simultaneously. It is one of the latest devices introduced in the last decade. In the harmonic scalpel, ultrasonic energy is transformed to mechanical energy at the active blade [5]. Its key benefits are exact dissection, reliable haemostasis, reduced lateral thermal spread, and charring.

The safety and efficacy of tissue dissection and artery sealing in colorectal surgery remain highly debatable. With the increased use of minimally invasive procedures in the colon and rectal surgery, energy devices for tissue dissection and vascular sealing have become widely used [6]. However, due to the convenience and cost-effectiveness of energy devices, traditional major vascular control devices such as clips and vascular staplers are gradually being phased out [7]. Instead, new gadgets are becoming more available, allowing us to perform procedures with reduced operating time and blood loss. This study compares conventional bipolar electrocautery and ultrasonic harmonic scalpel in CRC surgeries.

## Materials and methods

Study design

We conducted a hospital-based comparative study at our tertiary care hospital after obtaining approval from the Institutional Ethics Sub-Committee (IESC) of Dr D.Y. Patil Medical College, Hospital & Research Centre, Pune, IND. The approval number assigned to our study is IESC/PG/2019/83.

Inclusion criteria

Patients of both genders and age groups above 40 years with proven colorectal malignancy of stages I and II were included in our study.

Exclusion criteria

Patients with stage III and stage IV CRC, post-neoadjuvant chemotherapy patients, and immunocompromised patients were excluded from our study.

Data collection

Fifty patients who fulfilled the inclusion and exclusion criteria were enrolled for the study. Socio-demographic data and clinical information were obtained from the patient by detailed history and examination. Clinical staging was done. Patients were subjected to complete workup, which includes blood investigations: blood group, complete haemogram, liver function tests, renal function tests, serum electrolytes, serum proteins, blood sugar levels, HbA1c, urine routine, serology for human immunodeficiency virus, hepatitis B surface antigen, prothrombin time, bleeding time, clotting time; Radiological investigations: chest X-ray, X-ray abdomen, ultrasound abdomen and pelvis, contrast-enhanced computed tomography abdomen and pelvis, magnetic resonance imaging abdomen and pelvis (if required); Cardiological assessment: electrocardiogram, 2D-echo and tumour markers: carcinoembryonic antigen, carbohydrate antigen 19-9. Tissue diagnosis was made by performing a colonoscopic biopsy and sent for histopathological examination. Information regarding the tumour such as type, stage, and grade was recorded. Patients were revised based on age, tissue diagnosis, and stage of the tumour and were randomized into two groups by the block randomization method. Group A includes 25 patients in whom bipolar electrocautery was the energy source used during the surgery. Group B includes 25 patients in whom ultrasonic harmonic scalpel was the energy source used during the surgery.

Appropriate surgery was done as per the clinical presentation, histopathological examination, and stage and grade of the disease at presentation. As decided by randomization planned procedure was done either by using bipolar electrocautery or ultrasonic harmonic scalpel. The surgical procedure varied among right hemicolectomy, left hemicolectomy, anterior resection, and abdominoperineal resection based on site, extent, and stage of the tumour. Based on the surgical procedure, a covering or an end colostomy was done when required. The resected tumour specimen is sent for histopathological examination, and information regarding lymph node involvement, lymphovascular invasion, perineural invasion, and surgical margins was recorded. Total operative time from skin incision to closure was noted. Intraoperative blood loss was quantified by gauze visual analogue and recorded. Postoperatively, patients from both groups received Inj. ceftriaxone 2 g intravenously every 12 hours for seven days, Inj. metronidazole 100 ml intravenously every 8 hours for five days, Inj. diclofenac sodium intravenously, and Inj. pantoprazole 40 mg intravenously. Complications, if any, like surgical site infection, perineal wound soakage, and anastomotic leak were recorded. The number of days in hospital was noted. Follow-up was done at regular intervals of three months. Follow-up was taken up to two years to check for any recurrence and colostomy complications (if any) - retracted stoma, parastomal hernia, etc.

Statistical analysis

The data collected for two years were analyzed using SPSS version 17.0 software (SPSS Inc., Chicago) and results tabulated. The mean and standard deviation of the age of the study groups were calculated. The patients’ gender, tissue diagnosis, and the surgical procedure performed were reported as percentages. The operative time, blood loss, and hospital stay after surgery were calculated in both the groups and independent t-tests were performed to compare bipolar electrocautery and harmonic scalpel. A p-value <0.05 was considered statistically significant. 

## Results

The mean age of the study group was 61.80 years (standard deviation of 10.20 years). Among the 50 study samples, 26 (52%) patients were males, and 24 (48%) patients were females, with a mean age of 61.80 ± 10.20 years. Among the study sample, the most common histopathological finding was adenocarcinoma rectum with 16 (32%) patients, followed by adenocarcinoma sigmoid with 14 (28%) patients. Twelve (24%) patients had adenocarcinoma ascending colon and eight (16%) patients had adenocarcinoma descending colon. Patients with carcinoma sigmoid colon and carcinoma proximal rectum underwent anterior resection (20). Patients with carcinoma distal rectum underwent abdominoperineal resection (10). Patients with carcinoma ascending colon underwent right hemicolectomy (12), and patients with carcinoma descending colon underwent left hemicolectomy (eight).

The mean operating time in minutes in Group A (electrocautery) was 175.6 ± 27.4 minutes and in Group B (harmonic scalpel) was 137 ± 26.7 minutes (Table 1). The harmonic scalpel group had a lower mean operating time, which was statistically significant (p < 0.001).

**Table 1:** Comparison of mean operating time in electrocautery and harmonic scalpel groups.

Groups	Number of patients	Mean operating time (minutes)	SD	p-Value
Group A (electrocautery)	25	175.6	27.4	<0.001
Group B (harmonic scalpel)	25	137	26.7	
Independent t-test, p value-significant				

The mean blood loss during surgery in Group A (electrocautery) was 271.80 ± 80.6 ml, and the mean blood loss during surgery in Group B (harmonic scalpel) was 184 ± 68.10 ml (Table 2). The mean blood loss in the harmonic scalpel group was statistically lower than in the electrocautery group (p < 0.001).

**Table 2:** Comparison of mean blood loss in electrocautery and harmonic scalpel groups.

Groups	Number of patients	Mean blood loss (ml)	SD	p-Value
Group A (electrocautery)	25	271.8	80.6	<0.001
Group B (harmonic scalpel)	25	184	68.1	
Independent t-test, p value-significant				

The mean hospital stay in Group A (electrocautery) was 12.3 ± 2.6 days, and the mean hospital stay in Group B (harmonic scalpel) was 9.7 ± 2.2 days (Table 3). The hospital stay was longer in the electrocautery group and was statistically significant (p < 0.001).

**Table 3:** Comparison of mean hospital stay in electrocautery and harmonic scalpel groups.

Groups	Number of patients	Mean hospital stay (days)	SD	p-Value
Group A (electrocautery)	25	12.3	2.6	<0.001
Group B (harmonic scalpel)	25	9.7	2.2	
Independent t-test, p value-significant				

## Discussion

The research was conducted in Dr. D.Y. Patil Medical College and Research Centre, Pimpri, Pune, among 50 patients with stage I and stage II CRCs. All patients who came to the general surgery out-patient department fulfilling the inclusion and exclusion criteria were enrolled for the study. The mean age of the study group is 61.80 years, with 36% of the patients within the age group 61-70. The oldest participant was 79 years old, and the youngest participant was 45 years old. In a study done by Nitsche et al. on the effectiveness of surgery with rising patient age in CRC, the mean age was identified to be 66 years among 569 patients [8]. The study group comprised 26 (52%) male patients and 24 (48%) female patients with more or less equal gender distribution. In a study done by Farin Amersi et al., the incidence of CRC is equal in both men and women worldwide [9].

In our study, 16 (32%) patients presented with carcinoma rectum, including six patients with carcinoma proximal rectum and 10 patients with carcinoma distal rectum. Fourteen (28%) patients presented with carcinoma sigmoid colon. Twelve (24%) patients presented with carcinoma ascending colon followed by eight (16%) patients presented with carcinoma descending colon. In a study done by Maglinte et al. on spatial distribution and detection of colon and rectal cancer, 33.8% of patients had rectal cancer, 24.9% patients had sigmoid cancer, 8.7% patients had ascending colon cancer, and 7.1% patients had descending colon cancer. The remaining 25.4% of patients had caecum cancer and transverse colon cancer [10].

Operative time was measured in minutes from incision to skin closure. The mean operating time in Group A (electrocautery) was 175.6 ± 27.4 minutes. The mean operative time in Group B (harmonic scalpel) was 137 ± 26.7 minutes. There was a significant difference in the operating time in minutes when a harmonic scalpel was used as the energy source, which was statistically significant with a p-value of <0.001. In a study done by Targarona et al., the mean operative time for laparoscopic colectomy using conventional electrocautery was 180 minutes, significantly higher than the mean operative time for laparoscopic colectomy using ultrasonic dissection, which was 120 minutes [11].

Blood loss during the surgery was measured in millilitres using the gauze visual analogue technique. The mean blood loss in Group A (electrocautery) was 271.8 ± 80.6 ml. The mean blood loss in Group B (harmonic scalpel) was 184 ± 68.1 ml. A significant difference was observed in blood loss during surgery when a harmonic scalpel was used as the energy source, which was statistically significant with a p-value of <0.001. In a study done by Morino M et al., comparing ultrasonic versus standard electric dissection in laparoscopic colorectal surgeries, the mean blood loss was 182.6 ml during electric dissection, and the mean blood loss was 140.8 ml using ultrasonic dissection. Intraoperative blood loss was significantly low during ultrasonic dissection compared to electric dissection with a p-value <0.032 [12].

Hospital stay was measured in days from the day of surgery until discharge. The mean hospital stay in Group A (electrocautery) was 12.3 ± 2.6 days. The mean hospital stay in Group B (harmonic scalpel) was 9.7 ± 2.2 days. There was a significant difference in hospital stay when a harmonic scalpel was used as the energy source, which was statistically significant with a p-value of <0.001. According to Federico Sista et al., the average hospital stay was eight days in patients who underwent hemicolectomy using a harmonic scalpel, and the average hospital stay was 11 days in patients who underwent hemicolectomy using conventional haemostasis [13]. In the two-year follow-up period after surgery, done at regular intervals of three months, patients were monitored for recurrence. The patients from both the groups received chemotherapy and radiotherapy as per stage and grade of the tumour. Eighteen subjects from Group A and 20 subjects from Group B followed up after surgery for two years. The rest of the subjects were lost in follow-up. None of the patients had recurrence during the period of follow-up. Our study has some limitations, including the small sample size and operator dependence. Further studies involving a larger number of patients with a single operating surgeon increase the validity of the results.

## Conclusions

Harmonic scalpel is a valuable tool in colorectal surgery. The current study found that the harmonic scalpel outperformed the bipolar cautery as an energy source during CRC surgeries in terms of operative time, blood loss, and hospital stay. However, more research is needed on cost-effectiveness and post-operative complications. Further research with a larger sample size will confirm the findings.
